# Chiral amino acid metabolomics for novel biomarker screening in the prognosis of chronic kidney disease

**DOI:** 10.1038/srep26137

**Published:** 2016-05-18

**Authors:** Tomonori Kimura, Kenji Hamase, Yurika Miyoshi, Ryohei Yamamoto, Keiko Yasuda, Masashi Mita, Hiromi Rakugi, Terumasa Hayashi, Yoshitaka Isaka

**Affiliations:** 1 Department of Nephrology, Osaka University Graduate School of Medicine, Box B6, 2-2 Yamada-oka, Suita, Osaka, 565-0871, Japan; 2 Graduate School of Pharmaceutical Sciences, Kyushu University, 3-1-1 Maidashi, Higashi-ku, Fukuoka 812-8582, Japan; 3 Shiseido Co., Ltd., 1-1-16 Higashi-shimbashi, Minato-ku, Tokyo 105-0021, Japan; 4 Department of Geriatric and General Medicine, Osaka University Graduate School of Medicine, Box B6, 2-2 Yamada-oka, Suita, Osaka, Japan 565-0871; 5 Department of Kidney Disease and Hypertension, Osaka General Medical Center, 3-1-56, Bandaihigashi, Sumiyoshi-ku, Osaka, 558-8558, Japan; 6 Department of Nephrology, Rinku General Medical Center, Izumisano Municipal Hospital, 2-23 Rinku-Orai Kita, Izumisano, Osaka 598-8577, Japan

## Abstract

D-Amino acids, the enantiomers of L-amino acids, are increasingly recognized as novel biomarkers. Although the amounts of D-amino acids are usually very trace in human, some of them have sporadically been detected in blood from patients with kidney diseases. This study examined whether multiple chiral amino acids would be associated with kidney functions, comorbidities, and prognosis of chronic kidney disease (CKD) by enantioselective analyses of all chiral amino acids with a micro-two-dimensional high-performance liquid chromatograph (2D-HPLC)-based analytical platform. 16 out of 21 D-amino acids were detected in plasma from 108 CKD patients in a longitudinal cohort. The levels of D-Ser, D-Pro, and D-Asn were strongly associated with kidney function (estimated glomerular filtration ratio), the levels of D-Ala and D-Pro were associated with age, and the level of D-Asp and D-Pro were associated with the presence of diabetes mellitus. D-Ser and D-Asn were significantly associated with the progression of CKD in mutually-adjusted Cox regression analyses; the risk of composite end point (developing to ESKD or death before ESKD) was elevated from 2.7- to 3.8-fold in those with higher levels of plasma D-Ser and D-Asn. These findings identified chiral amino acids as potential biomarkers in kidney diseases.

Chronic kidney disease (CKD) is a critical health problem^1^. CKD patients are widely prevalent, and the number of end-stage kidney disease (ESKD) patients, who need to receive cost-prohibitive kidney replacement therapy, is still increasing^2^. Additionally, the risk of cardiovascular events and death increases with the progression of CKD stages^3^,^4^,^5^. The critical issues in the management of CKD are, therefore, the early diagnosis of CKD and prevention of CKD patients from the progression to ESKD.

D-Amino acids, the enantiomers of L-amino acids, are increasingly recognized as physiologically active molecules as well as potential biomarkers. Their distribution and regulation are totally different from those of L-forms^6^. Although the amounts of D-amino acids are usually very trace in humans, some of the D-amino acids indeed have physiological functions; D-Ser, for example, is known to function as a key neuro-modulator in brain^7^,^8^,^9^,^10^,^11^,^12^,^13^.

Several reports suggest the potential relationships between the amounts of D-amino acids and kidney function. These lines of reports indicate that the levels of some D-amino acids are correlated with serum creatinine in patients with kidney dysfunction^14^,^15^,^16^,^17^,^18^. The serum level of D-Ser was also shown to increase after ischemia/reperfusion kidney injury in mice^18^.

These lines of evidence indicate that D-amino acids are promising candidates as biomarkers for several diseases including kidney diseases, however, no comprehensive study has been conducted due to the lack of comprehensive analytical platform of D-amino acids. Recently, a highly selective two-dimensional high-performance liquid chromatography (2D-HPLC) combined with highly-sensitive fluorescence derivation has shown to be effective in the enantioselective analyses of all proteinogenic amino acids^19^,^20^,^21^. In this study, we utilized this 2D-HPLC-based analytical platform and performed the chiral amino acids focused-metabolic profiling of a longitudinal cohort, which was composed of referred CKD patients, with the goal of identifying the D-amino acids which predicts the earlier deterioration of kidney function.

## Results

### A chiral amino acid metabolic profiling of the cohort

We performed a chiral amino acids metabolomic profiling on a longitudinal cohort, which consisted of referred CKD patients. Data were generated from 108 participants with median follow-up period of 4.3 years (interquartile ranges, 2.4–5.5). None of the participants were lost to follow. The baseline characteristic is shown in Table 1. Among these patients, 58 started kidney replacement therapy and 15 died, including 4 who died before initiating kidney replacement therapy.

**Figure 1:**
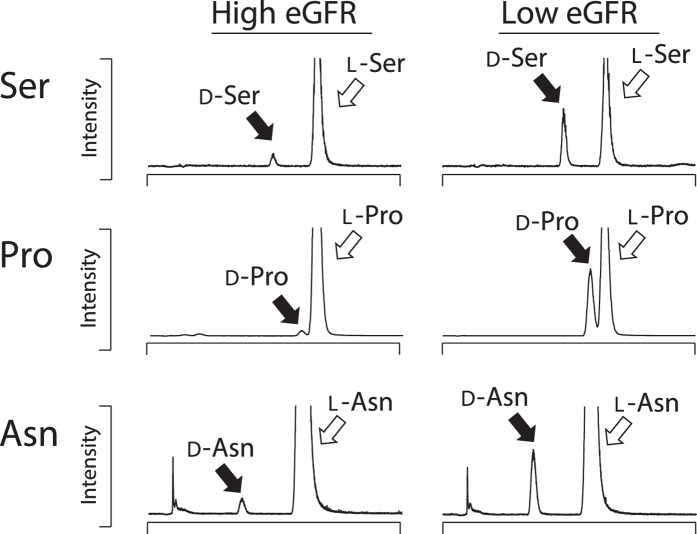
Detection of 4-fluoro-7-nitro-2,1,3-benzoxadiazole (NBD)-labeled amino acid enantiomers in the plasma of patients with chronic kidney disease using a 2D-HPLC-MS/MS system. The detailed MS/MS conditions were described in the Method section.

We applied the 2D-HPLC determination for the metabolomic profiling of this cohort. This system enables us to detect all proteinogenic amino acid enantiomers ranging from ca. 1 fmol to 100 pmol quantitatively with chiral selectivity without severe interference from intrinsic substances^19^,^20^,^21^. The relative standard deviations of between-run precision (coefficient of variation for each amino acid determined by 4 repeated analyses) were 1.10–8.19%. Concerning the repeatability in analyzing human plasma samples, duplicate analyses were performed using several samples, and almost the same results were obtained. Several D-form amino acids were observed in the plasma of CKD patients by the present enantioselective 2D-HPLC analyses (Fig. 1 and Table S1); D-Ser, D-Ala, D-Pro, and D-Asn were detected in most of the patients (89–100%), D-Asp, D-Lys, D-*allo*-Thr, D-Glu, D-Arg, and D-His were detected in 10–40% of patients, and D-Leu, D-Phe, and D-*allo*-Ile were detected sporadically (<10% of patients). Of note, *allo*-Thr detected in this cohort solely consisted of D-*allo*-Thr. In living organisms, conversion of “only” alpha carbon predominantly occurs, which results in the formation of “D-*allo*-forms” from L-Thr and L-Ile. Therefore, the abundance of D-*allo*-forms is higher than D-forms concerning these two amino acids.

In summary, 13 D-amino acids were detected in CKD patients’ plasma from this cohort by the present 2D-HPLC system. One patient, who was uremic on arrival and was excluded from this study because dialysis therapy had immediately been initiated, showed an interesting profile of D-amino acids (Table S2). This patient’s levels of D-amino acids were extremely high. In addition, 3 D-amino acids which were hardly detected in this cohort (D-Val, D-Ile, and D-Gln) were also detected in this patient’s plasma (Table S2). Together, the 2D-HPLC system enabled us to detect 16 out of 21 D-amino acids in human plasma (Table S1).

### Association of chiral amino acids with clinical parameters

We assessed the correlations between clinical parameters and baseline concentrations of chiral amino acids. The levels of D-Ser, D-Pro, and D-Asn showed strong reverse-association with estimated glomerular filtration ratio (eGFR), whereas others (D-Ala, D-Asp, and D-*allo*-Thr) also showed weak correlation (Fig. S1). Noteworthy is that the level of L-Ser showed positive correlation with eGFR, and thus, the ratio of D/L-Ser showed further strong correlation with eGFR.

Because the relationships between the levels of D-amino acids and such comorbidities of CKD as age and diabetes mellitus were reported^22^,^23^, we explored those associations. Age, a main factor for kidney function, was correlated with the levels of D-Ala, D-Pro, L-Ser, and weakly with L-Ile (Fig. S2). With regards to the presence of diabetes, a poor prognostic factor for CKD patients, the levels of D-Asp and D-Pro were lower, and the level of L-Ile being higher, in diabetic patients compared with those in non-diabetic ones (Fig. S3). These findings indicate that the ratios of D/L-amino acids were variably related with kidney function and morbidity factors for CKD.

### Association of chiral amino acids with worsening of kidney function

**Figure 2:**
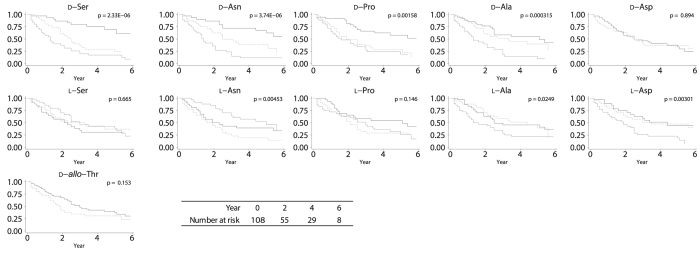
Kaplan-Meier curves of D/L-amino acids for the prognosis of kidney disease. Patients with first (thick line), second (dotted line), and third (thin gray line) tertile of levels of amino acids were subjected to these analyses.

Kaplan-Meier curve analyses demonstrated that patients with higher levels of all highly-detected D-amino acids (D-Ser, D-Ala, D-Pro, and D-Asn) reached the composite outcome (a combination of end-stage kidney disease requiring replacement therapy and all-cause of death) more frequently compared with the other group of patients (Figs 2 and S4). When we divide the patients by kidney function, these separations for composite outcome were seen in D-Ala and D-Pro in patients with eGFR more than 20 mL/min/1.73m^2^, although the similar results were also seen in D-Ser and D-Ala (Fig. S5). Some of less-detected D-amino acids (D-Lys, D-Leu, and D-*allo*-Ile), as well as some L-amino acids (L-Asp, L-Glu, L-Ala, L-Trp, and L-Lys), also separated the kidney prognosis (Figs 2 and S4).

We fitted multiple Cox regression analyses to assess the association between highly-detected D-amino acids with composite outcome. The Holm-corrected multiple testing revealed that the levels of all highly-detected D-amino acids (D-Ser, D-Ala, D-Pro, and D-Asn) had predictive values on the composite outcome (Table S3). Patients in top tertile of the levels of D-Ser and D-Asn had 2.7- to 3.7- fold higher adjusted hazard ratios for composite outcome after adjustment for kidney function and urinary protein level compared with those in lowest tertile (Table 2, Model 1). These findings were similar when we further adjusted with other comorbidities such as age and the presence of diabetes, which had some effect on the levels of D-amino acids (Table 2, Model 2). Thus, these metabolites were associated with the progression of kidney diseases in CKD patients.

## Discussion

We performed a chiral amino acid metabolomic profiling of a cohort consisted of advanced CKD patients, and detected 16 D-amino acids in plasma by utilizing 2D-HPLC system. The levels of D-amino acids were associated with kidney function and the presence of co-morbidities, such as older age and diabetes mellitus. Furthermore, the higher levels of D-Asn and D-Ser were associated with the progression to ESKD; the risk of developing to the ESKD was elevated from 2.7- to 3.8-fold in those with higher levels of plasma metabolites. Although the physiology and metabolism of D-amino acids largely remain mystery, the present study demonstrated clinical potential of D-amino acids as biomarkers in CKD.

Our 2D-HPLC system delineated previously underappreciated association of D-Asn with kidney prognosis. The measurable levels of some D-amino acids have sporadically been detected in blood from patients with kidney dysfunction^14^,^15^,^16^,^17^, however, incompletely due to the trace amount of D-amino acids in the samples. Up to our knowledge, the physiological and clinical significance of D-Asn have not assessed previously because of the difficulty of measurement. D-Asx (D-Asx = D-Asp + D-Asn) was detected in the serum from kidney patients^16^, however, the levels of these two chiral amino acids were not determined separately in the conventional methods. With accurate 2D-HPLC-based analytical platform and the longitudinal information, we demonstrated that the increased levels of D-amino acids were associated with worse prognoses of CKD patients. The present 2D-HPLC-based analytical platform will provide the strong technical basis for further chiral amino acid metabolomics.

Though the mechanisms why a subset of D-amino acids accumulate in CKD patients are largely unknown, kidneys may play essential roles to keep the levels of D-amino acids very low in blood by excretion and metabolism. D-Ser is known to increase its level in blood, and to decrease its level in urine, in response to acute kidney injury in mice^18^. Genetic ablation study revealed that this increase was at least partially due to decreased activity of D-amino acid oxidase (DAO), but not by activation of serine racemase (D-Ser synthetic enzyme from L-Ser)^18^. DAO, which is mainly distributed in proximal tubules (main lesions insulted by acute and chronic kidney injury)^24^, metabolizes D-Ser upon reabsorption. A high level of DAO was detected in the urine from patients with chronic kidney insufficiency^17^, while ischemia/reperfusion injury decreased DAO activity in rodent kidneys^18^,^24^. These facts may indicate that DAO activity is decreased in injured kidneys due to its excretion upon damages. On the other hand, kidney proximal tubules have a potential to reabsorb D-Ser, whose effect is basically limited because the overwhelming amount of L-Ser competes this process. Damages on proximal tubules may further decrease the reabsorption of D-Ser. Furthermore, D-Ser, in itself, is known to have nephrotoxicity^25^,^26^; its increase in blood by kidney injury may in turn exacerbate the worsening course of kidney injury. Therefore, acute and chronic kidney injuries on proximal tubules may lead to higher level of D-Ser in blood through the decrease of DAO activity and reabsorption of D-Ser, which, in turn, may further worsen kidney function.

Although the physiological roles of D-amino acids are largely unknown, especially in D-Asn, some associations between some D-amino acids and clinical conditions are reported. D-Ser is known to be present at a high level in the forebrain area including the cerebrum and hippocampus^27^, and functions as an endogenous ligand of *N*-methyl-D-aspartate receptor (NMDAR)^28^ to modulate synaptic plasticity^8^,^9^,^10^. The fact that the level of D-Ser increases in spinal fluid of patients with Alzheimer’s disease^29^ and in hippocampus in aged rat^30^ may indicate that D-Ser plays a critical role in cognitive ability. The aberrant metabolism of D-Ser seen in this study may be associated with cognitive disability in CKD patients^31^. D-Asp, which is widely distributed in the body especially in neuroendocrine and endocrine tissues^6^,^32^,^33^,^34^, is also associated with aging and stress^23^,^34^. D-Ala is known to be distributed in pituitary gland and in pancreas^7^,^22^, while relatively high concentration of D-Pro is present in pituitary gland and pancreas of mice^35^. The level of D-Ser increased in the brain of streptozotocin-treated mice (type 1 diabetes model)^13^. Insulin is considered to have the potential to decrease D-Ser level because treatment with insulin on streptozotocin-treated mice restored its elevated level. Our additional observation that the higher level of L-Ile, which is associated with the development of diabetes^36^ and the potential activation of the mammalian target of rapamycin^37^, in patients with diabetes strongly suggests that L-Ile may also indicate a potential benefit to detect co-morbidity and predict diabetes-associated complications. Although kidney tubules handle the metabolism of D-amino acids, other organs also participate in their metabolism in yet mysterious ways. Therefore, the levels of D-amino acids might not be a simple reflection of kidney tubular injury like recently-emerging tubular injury markers such as neutrophil gelatinase-associated lipocalin (NGAL)^38^ or kidney injury molecule-1 (KIM-1)^39^; instead, D-amino acids may add more important information like prognosis or the cormobidities. D-amino acids also harbor the potentials to be biomarkers for several diseases and physiological conditions.

This study has some limitations. Although the present study was strengthened by the longitudinal observation, the limitations exist on relatively small number of the cohort and the dependence only on the baseline characteristics. We performed power analyses using a type I error of 5% and 80% power. These analyses revealed that 25 or 38 patients would be needed for D-Asn (hazard ratio, 3.07) or D-Ser (hazard ratio, 2.49) of the selected metabolites in the prediction set, respectively. Therefore, we had enough sample size to determine the association between composite outcomes and these metabolites, although there remains a potential that the effects of D-Ala, D-Pro, and other undetected metabolites might be underestimated. As aforementioned, we also have no mechanistic data to demonstrate the reasons for the changes of the levels of these chiral amino acids, and this needs to be addressed in the future study. Further studies are warranted to test whether measuring these chiral amino acids in addition to traditional markers would benefit CKD patients.

In summary, our chiral amino acid metabolomics revealed a subset of D-amino acids as potential biomarkers for kidney diseases. Given the importance of kidney on D-amino acids handling, the present study provides important direction of biomarker mining in kidney diseases.

## Methods

### Study population and samples

We prospectively enrolled 118 consecutive patients with CKD stages 3, 4 and 5, who were not on dialysis, from a single nephrology department at Rinku General Medical Centre between August 2005 and January 2009. This was a period when the movement of early referral of CKD patients had just launched. Baseline blood samples from patients after an overnight fast were collected into plastic tubes to prepare plasma. Patients with insufficient blood samples were excluded beforehand. This study was approved by the institutional ethics committees of Rinku General Medical Centre and Osaka General Medical Centre, and was adherent to the Declaration of Helsinki.

Baseline inclusion criteria included age less than 90 years, no complication of malignancy, and no active infection. Patients with incomplete baseline data (n = 2) or insufficient amount of plasma samples (n = 4), and started kidney replacement therapy within 1 month after the enrolment (n = 4) were excluded. The study was approved by the institutional ethics committees of Rinku General Medical Centre and by the Osaka General Medical Centre, and all patients provided written informed consent to participate in the study. Kidney function was evaluated from baseline data at the first visit to our outpatient clinic using the estimated glomerular filtration rate (eGFR) based on newly developed equation for Japanese population^40^. The formula is as follows: eGFR = 194 × serum creatinine (SCr)^−1.094^ × age^−0.287^, where age is in years, SCr is in mg/dL and the glomerular filtration rate (GFR) is in mL/min/1.73 m^2^ body surface area. The product of this equation for women was multiplied by a correction factor of 0.739. Serum creatinine was measured by enzymatic methods in the same laboratory. Random urine samples (10 mL) were also collected at baseline to measure the ratio of urinary protein to creatinine. Other baseline variables included age, sex, diabetes defined according to the International Classification of Diseases, Tenth Revision (ICD-10) codes E10-E14, systolic blood pressure, diastolic blood pressure, hemoglobin, and use of renin-angiotensin system inhibitor, beta-blocker, and calcium blocker. Origin of kidney diseases were diagnosed based on biopsy or clinical suspicion.

The primary end point in this study was a composite of end-stage kidney disease (ESKD) requiring kidney replacement therapy and all-cause of death. Patients received regular follow-up care in the outpatient ward. Data were collected from source documentation at the end of 2011. The baseline and follow-up data were collected from hospital medical records and discharge abstracts, outpatient visit records, contact with primary and dialysis care physicians and death certificates. End points were validated by at least two physicians. The follow-up of patients is available with accuracy because (i) this facility is the central hospital of the southern region of Osaka prefecture, and there is no other central hospital in this region, and (ii) regional partnership with the primary and dialysis care physicians has been well-developed.

### Sample preparation

Sample preparation from human plasma was performed as previously described with modification^19^. In brief, 20-fold volumes of methanol were added to the plasma and mixed vigorously. After centrifugation, an aliquot (10 μL of the supernatant obtained from the methanol homogenate) was placed in a brown tube was dried under reduced pressure. To the residue, 20 μL of 200 mM sodium borate buffer (pH 8.0) and 5 μL of fluorescence labeling reagent (40 mM 4-fluoro-7-nitro-2,1,3-benzoxadiazole (NBD-F) in anhydrous MeCN) were added, then heated at 60 °C for 2 min. An aqueous 0.1% (v/v) TFA solution (75 μL) was added to stop the reaction, and 2 μL of the reaction mixture was subjected to 2D-HPLC.

### Determination of amino acid enantiomers by 2D-HPLC

The enantiomers of amino acids were quantified using the micro 2D-HPLC platform, as previously described^20^,^21^. In brief, the NBD-derivatives of the amino acids were separated using a reversed-phase column (monolithic ODS column, 0.53 mm i.d. × 1000 mm; provided by Shiseido, Tokyo, Japan) with the gradient elution using aqueous mobile phases containing 5–35% MeCN, 0–20% THF, and 0.05% TFA. Column temperature was set at 45 °C, and the flow rate of mobile phase was 25 μL/min. In order to separately determine the D- and L-forms, the fractions of the target amino acids were automatically collected using a multi-loop valve, and transferred to the enantioselective column (KSAACSP-001S or Sumichiral OA-3200, 1.5 mm i.d. × 250 mm; self-packed. Materials were obtained from Shiseido and Sumika Chemical Analysis Service, Osaka, Japan, respectively). For the measurements of Ile and Thr, which has 4 stereoisomers (L-, D-, L-allo-, and D-allo-forms), we separated (L- and D-) forms and diastereomer (L-allo- and D-allo-) forms by the reversed-phase mode in the first dimension (these diastereomers are separable by the reversed-phase mode). Then their enantiomers (L- and D-, L-allo- and D-allo-) were separated in the second dimension by the enantioselective column. The mobile phases are the mixed solution of MeOH-MeCN containing citric acid (0–10 mM) or formic acid (0–4%), depending on the retention of amino acids. The fluorescence detection of the NBD-amino acids was carried out at 530 nm with excitation at 470 nm. The retention time of NBD-amino acids were determined by authentic amino acid enantiomers, and the amounts were determined by the calibration lines. All the quantitative data were obtained by the fluorescence detection. In the second dimension, HPLC-MS/MS was used to confirm the presence of D-amino acids in the real biological matrices. API-5000 and Triple Quad 5500 (ABSciex, Framingham, MA, USA) were used as MS/MS instruments. The conditions for NBD-Ser were as follows: mode, positive-ion mode; precursor ion, 269; product ion, 252; ionspray voltage, 5500 V; ion source gas 1, 40 psi; ion source gas 2, 60 psi; curtain gas pressure, 20 psi; collision gas pressure, 12 psi; turbo gas temperature, 600 °C; declustering potential, 116 V; entrance potential, 10 V, collision cell exit potential, 24 V; and collision energy, 31 eV. The conditions for NBD-Pro were as follows: mode, positive-ion mode; precursor ion, 279; product ion, 262; ionspray voltage, 5500 V; ion source gas 1, 30 psi; ion source gas 2, 70 psi; curtain gas pressure, 20 psi; collision gas pressure, 11 psi; turbo gas temperature, 700 °C; declustering potential, 101 V; entrance potential, 10 V, collision cell exit potential, 18 V; and collision energy, 21 eV. The conditions for NBD-Asn were as follows: mode, positive-ion mode; precursor ion, 296; product ion, 193; ionspray voltage, 5500 V; ion source gas 1, 80 psi; ion source gas 2, 60 psi; curtain gas pressure, 10 psi; collision gas pressure, 5 psi; turbo gas temperature, 700 °C; declustering potential, 96 V; entrance potential, 10 V, collision cell exit potential, 12 V; and collision energy, 23 eV. The collision gas used for the selected reaction monitoring (SRM) mode was nitrogen gas.

### Statistics

Continuous variables are presented as medians and ranges or interquartile ranges (IQR). Discrete data are given as counts and ratios (%). Metabolites are presented as median (quantiles). Correlations were assessed using Spearman rank regression analysis. We constructed multivariate Cox proportional hazard models to assess the prognostic roles of metabolites by adjustment with potential confounders for progression to ESKD and clinically relevant baseline characteristics. Multiple comparison of the hazard models were performed with the Holm method to avoid Type I error. Log-rank test was used to assess the equality of survival distribution stratified by the median values. Statistical significance was defined as *P* < 0.05. Statistical analyses were performed using STATA statistical software version 11 (STATA Corporation, College Station, TX, USA).

## Additional Information

**How to cite this article**: Kimura, T. *et al*. Chiral amino acid metabolomics for novel biomarker screening in the prognosis of chronic kidney disease. *Sci. Rep*. **6**, 26137; doi: 10.1038/srep26137 (2016).

## Supplementary Material

Supplementary Information

## Figures and Tables

**Table 1:** Baseline characteristics of the patients.

Characteristic	All population (n = 108)
Age (yr)	65.3 ± 10.9
Male gender (%)	75.0
eGFR (mL/min/1.73 m^2^)	21.0 ± 12.4
Mean blood pressure (mmHg)	95.1 ± 12.9
Systolic blood pressure (mmHg)	139.1 ± 21.7
Diastolic blood pressure (mmHg)	73.2 ± 11.7
Hemoglobin (g/dL)	11.0 ± 1.9
Urinary protein (g/gCre)	2.8 ± 3.8
Origin of disease (%)	
Diabetes mellitus	30.6
Chronic glomerular nephritis	23.1
Others	45.4
Use of ACEi and/or ARB (%)	68.8
Use of beta-blocker (%)	32.4
Use of calcium blocker (%)	67.6

Values are described as mean ± SD or %.

eGFR, estimated glomerular filtration ratio; ACEi, angiotensin converting enzyme inhibitor; ARB, angiotensin receptor blocker.

**Table 2:** Cox regression analysis of D-amino acids for the risk of composite outcomes.

D-Amino acid	D-Asn	D-Ser	D-Ala	D-Pro
Unadjusted model				
First tertile	1.00 (Reference)	1.00 (Reference)	1.00 (Reference)	1.00 (Reference)
Second tertile	2.37(1.14–4.94)	3.27(1.50–7.13)	1.38(0.72–2.67)	2.56(1.32–4.97)
Third tertile	5.06(2.49–10.29)	5.68(2.69–11.99)	3.11(1.67–5.97)	3.05(1.57–5.93)
Model 1				
First tertile	1.00 (Reference)	1.00 (Reference)	1.00 (Reference)	1.00 (Reference)
Second tertile	1.82(0.85–3.94)	1.62(0.70–3.73)	0.78(0.4–1.53)	1.51(0.76–3.00)
Third tertile	3.76(1.74–8.09)	2.81(1.23–6.37)	1.28(0.66–2.50)	1.38(0.68–2.79)
Models were developed by adjusting eGFR and urinary protein level.				
Model 2				
First tertile	1.00 (Reference)	1.00 (Reference)	1.00 (Reference)	1.00 (Reference)
Second tertile	1.54(0.69–3.44)	1.30(0.54–3.09)	0.98(0.48–2.00)	1.60(0.80–3.21)
Third tertile	3.07(1.30–7.26)	2.49(1.00–6.19)	1.41(0.71–2.79)	1.57(0.79–3.11)

Models were developed by adjusting eGFR, urinary protein level, diabetes, age, sex, hemoglobin level, mean blood pressure, history of cardiovascular events, and hypertension medication use.

Values are described as hazard ratio (95% CI).
