# Supplementary material for: Chiral amino acid metabolomics for novel biomarker screening in the prognosis of chronic kidney disease
Source: Sci Rep. 2016 May 18;6:26137. doi: 10.1038/srep26137 (PMC4870615; doi:10.1038/srep26137)
Supplement: Supplementary Information [file srep26137-s1.pdf]

Supplemental Information

Chiral amino acid metabolomics for novel biomarker screening in the prognosis of chronic kidney disease

Tomonori Kimura, Kenji Hamase, Yurika Miyoshi, Ryohei Yamamoto, Keiko Yasuda, Masashi Mita, Hiromi Rakugi, Terumasa Hayashi, and Yoshitaka Isaka

5 Supplemental figures
3 Supplemental tables

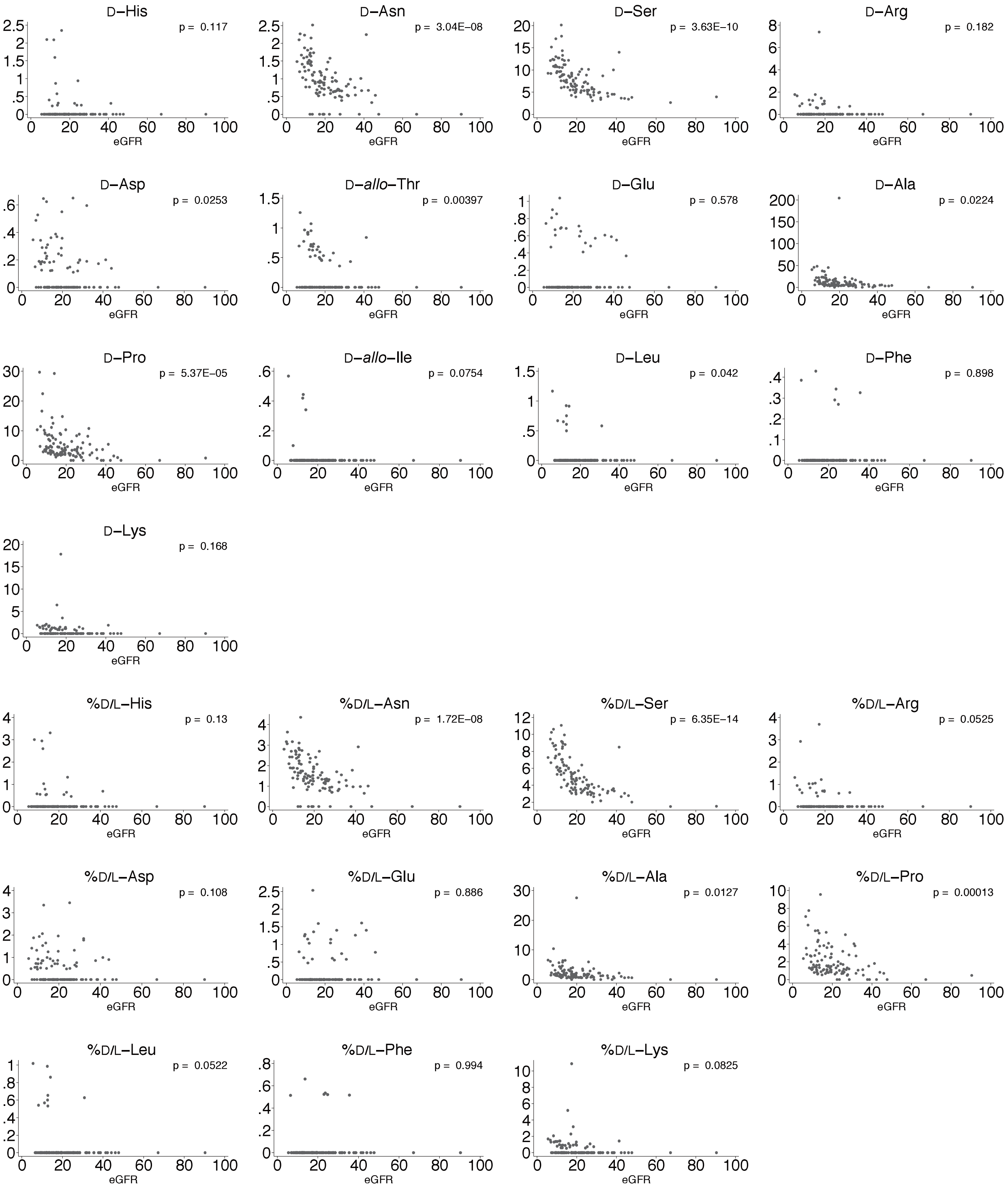

Figure S1. The relationships between D/L-amino acids and eGFR.
The values of amino acids were described as μmoL/L. %D/L, D/L ratio.

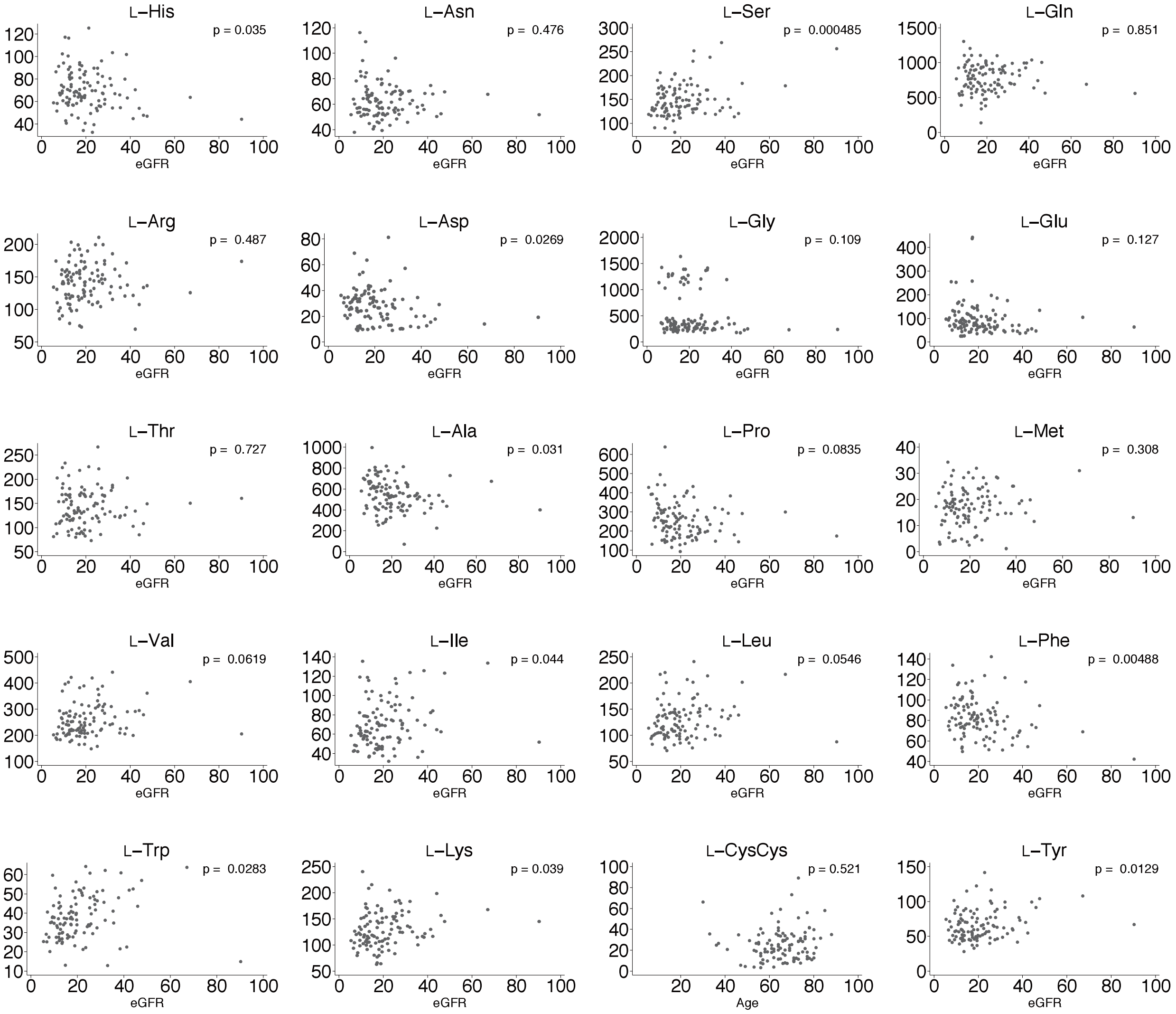

Figure S1, continued.

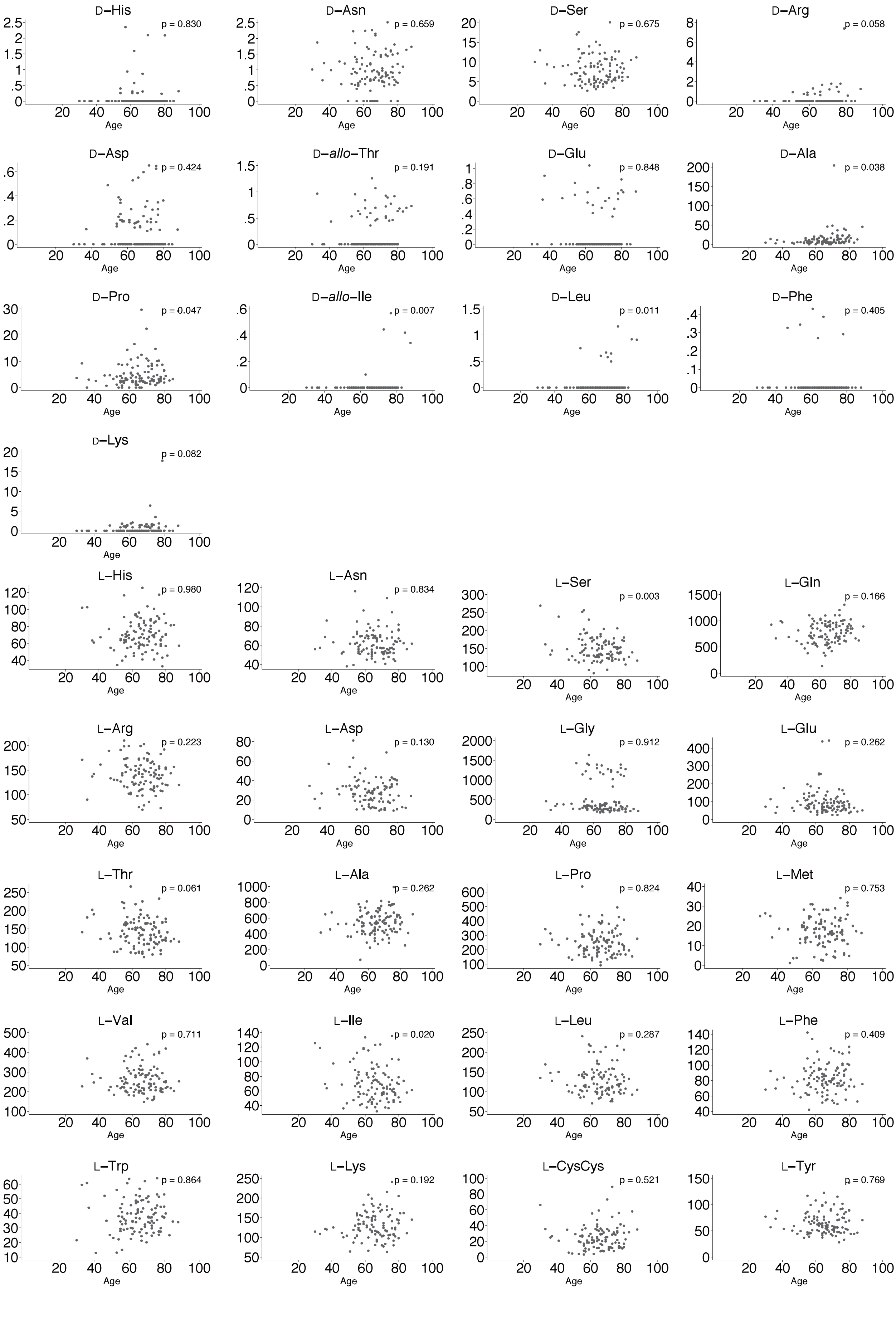

Figure S2. The relationships between D/L-amino acids and age. The values of amino acids were described as μmoL/L.

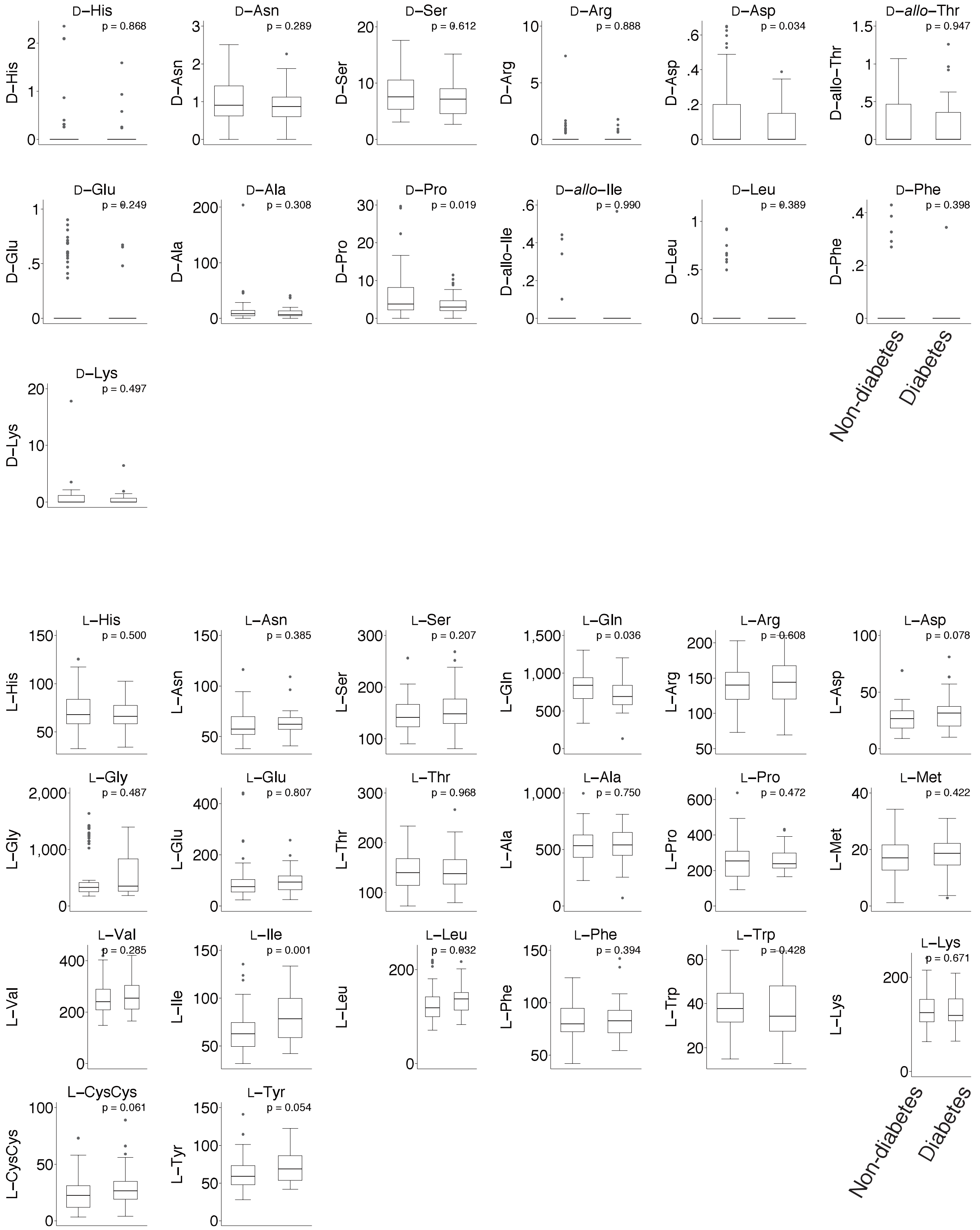

Figure S3. The relationships between D/L-amino acids and the complications of diabetes mellitus. The values of amino acids were described as μmoL/L.

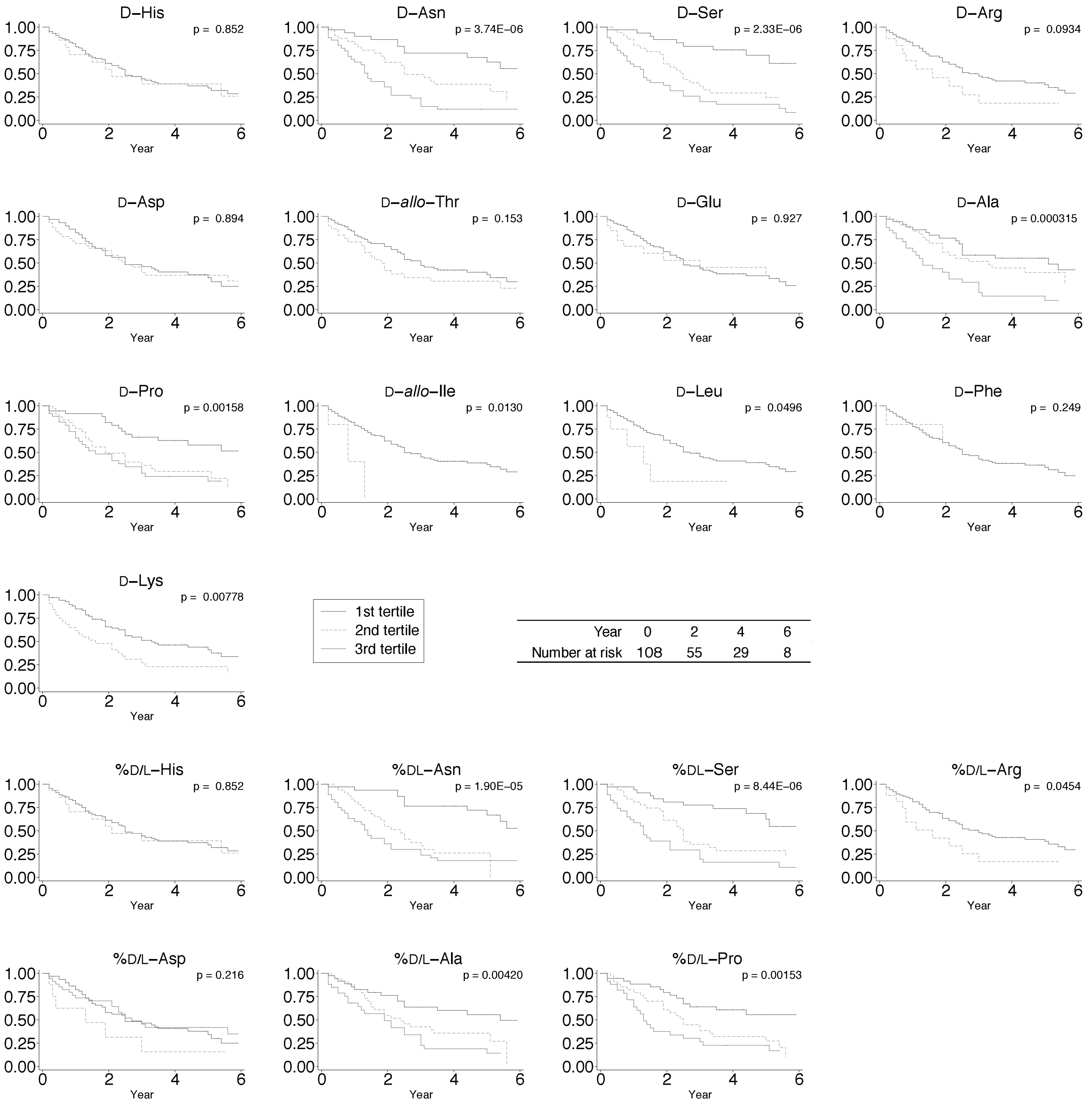

Figure S4. Kaplan-Meier curves of D/L-amino acids for the prognosis of kidney disease. Patients with first (thick line), second (dotted line), and third (thin gray line) tertile of levels of amino acids were subjected to these analyses.

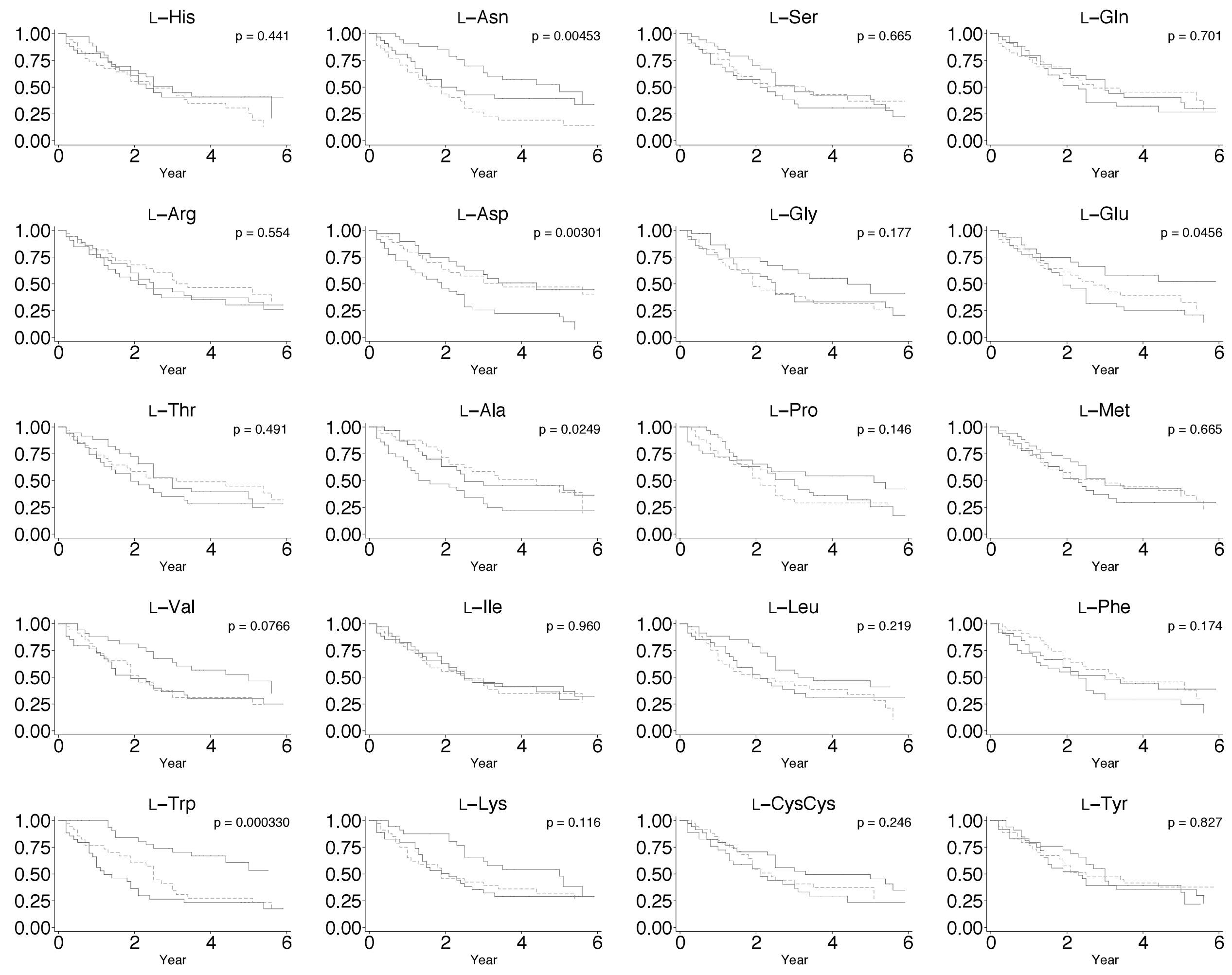

Figure S4, continued.

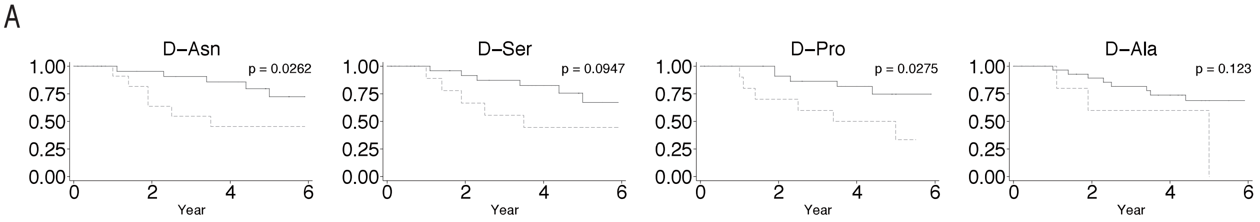

Year	0	2	4	6
Number at risk	42	27	19	6

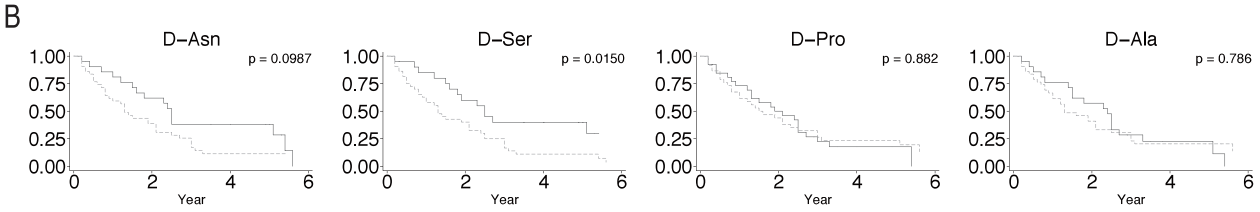

Year	0	2	4	6
Number at risk	66	28	10	2

Figure S5. Kaplan-Meier curves of D/L-amino acids divided by kidney function for the prognosis of kidney disease. Patients with higher (thick line) and lower (dotted line) of levels of amino acids were subjected to these analyses.

Table S1. The levels of D/L-amino acids of this cohort.

Amino acid	Detection, %		Value, median (IQR)	
	D-	L-	D-	L-
Ser	100	100	7.33 (5.31 - 9.78)	143 (126 - 169)
Ala	94.4	100	7.1 (4.5 - 14.0)	536 (435 - 630)
Pro	92.6	100	3.5 (2.1 - 6.4)	249 (190 - 308)
Asn	88.9	100	0.90 (0.62 - 1.34)	61 (53 - 69)
Asp	40.7	100	0 (0 - 0.19)	27 (19 - 34)
Lys	31.5	100	0 (0 - 0.92)	124 (107 - 153)
allo-Thr	27.8	0	0 (0 - 0.45)	0 (0 - 0)
Glu	20.4	100	0 (0 - 0)	78 (59 - 111)
Arg	15.7	100	0 (0 - 0)	140 (120 - 160)
His	13.9	100	0 (0 - 0)	68 (59 - 81)
Leu	8.3	100	0 (0 - 0)	122 (102 - 144)
Phe	5.6	100	0 (0 - 0)	81 (72 - 93)
allo-Ile	4.6	0	0 (0 - 0)	0 (0 - 0)
Met	0.0	100	0 (0 - 0)	17 (13 - 22)
Trp	0.0	100	0 (0 - 0)	37 (30 - 46)
Gln	0	100	0 (0 - 0)	773 (637 - 930)
Gly	-	100	-	336 (257 - 456)
Thr	0	100	0 (0 - 0)	138 (115 - 167)
Val	0	100	0 (0 - 0)	243 (211 - 291)
Ile	0	100	0 (0 - 0)	66 (54 - 82)
CysCys	0	100	0 (0 - 0)	24 (14 - 34)
Tyr	0	100	0 (0 - 0)	60 (49 - 77)

Values are described as µmoL/L. Note that Gly does not have a chirality and its value is described as L-form. IQR, interquartile ranges.

Table S2. D-Amino acids profile of a patient.

Compound	D-	L-	D/L (%)
His	0	54.55	0
Asn	2.39	31.82	7.52
Ser	17.13	70.15	24.42
Gln	0.58	514.79	0.11
Arg	2.50	95.08	2.63
Asp	0.14	12.43	1.11
Gly	-	831.58	-
allo-Thr	0	0	-
Glu	0.12	29.34	0.40
Thr	0	51.55	0.00
Ala	82.83	206.39	40.13
Pro	39.41	129.84	30.35
Met	0	9.73	0
Val	0.33	107.41	0.30
allo-Ile	1.06	0	-
Ile	0.04	25.49	0.14
Leu	1.56	45.28	3.44
Phe	0.35	67.29	0.52
Trp	0	11.62	0
Lys	5.12	66.81	7.67
CysCys	0	44.67	0
Tyr	0	33.71	0

Values are described as μmoL/L.

Table S3. Holm-adjusted Cox regression analyses for the composite outcome

D-Amino acid	Unadjusted *P*	Adjusted *P*
D-Asn	3.74E-06	1.50E-05
D-Ser	2.33E-06	9.32E-06
D-Ala	3.15E-04	1.26E-03
D-Pro	1.58E-03	6.32E-03

Crude P values for trend were determined by Cox regression analyses. Adjustment for crude *P* values were performed by Holm-method (adjusted *P*).
